# Experience of CBT in adults with ADHD: a mixed methods study

**DOI:** 10.3389/fpsyt.2024.1341624

**Published:** 2024-06-19

**Authors:** Sandy William, Matthew Horrocks, Jemma Richmond, Charlotte L. Hall, Blandine French

**Affiliations:** ^1^ School of Psychology, University of Nottingham, Nottingham, United Kingdom; ^2^ School of Health Sciences, University of Nottingham, Nottingham, United Kingdom; ^3^ Institute of Mental Health, University of Nottingham, Nottingham, United Kingdom; ^4^ School of Social Sciences and Humanities, University of Suffolk, Ipswich, United Kingdom

**Keywords:** attention-deficit hyperactivity disorder (ADHD), cognitive-behavioural therapy (CBT), adapted CBT, psychotherapy, interviews

## Abstract

**Introduction:**

The National Institute for Health and Care Excellence (NICE) recommends Cognitive-Behavioural therapy (CBT) as the psychotherapeutic treatment of choice for adults with Attention Deficit Hyperactivity Disorder (ADHD) in the UK. However, the literature often refers to adapted CBT programs tailored for ADHD and provides limited insight into how adults with ADHD experience and perceive this form of treatment in routine clinical practice.

**Methods:**

This mixed-methods study aims to explore ADHD individuals’ experience and perception of CBT delivered in routine clinical practice, to gain a better understanding of this treatment’s helpfulness and perceived effectiveness.

**Results:**

A survey (n=46) and semi-structured in-depth interviews (n=10) were conducted to explore the experience of CBT and its perceived effectiveness in managing ADHD. The interviews were analysed using thematic analysis and the survey was synthesised using descriptive narratives. The thematic analysis highlighted three key themes: difficulties with the CBT framework, difficulties with CBT therapists, and consequences of CBT. The survey highlighted similar findings. Participants described the CBT framework as, generic, rigid, and too short, and described the CBT therapist as unspecialised, unempathetic, and not sufficiently adapting CBT to ADHD-related difficulties.

**Discussions:**

Overall, participants found non-adapted, generic CBT in the UK to be unhelpful, overwhelming, and at times harmful to their mental well-being. Therefore, it is necessary for clinical bodies in the UK, while following the indicated NICE guidelines, to be mindful of adapting CBT delivery of CBT, to be most effective for people with ADHD and to mitigate potential harm.

## 1 Introduction

Attention-Deficit Hyperactivity Disorder (ADHD) is a neurodevelopmental condition characterised by symptoms of persistent inattention and/or hyperactivity-impulsivity, that causes clinical impairment in academic and social functioning (1) affecting approximately 5% of children (2) and 2.5% of adults (3). While this suggests that ADHD attenuates over time, the prevalence of symptomatic adults is estimated to be 6.7% (3).

ADHD is centrally a disorder of impaired executive functions (EFs) creating a devastating effect on self-regulation (4), inhibition, planning and working memory (5). These impairments impact many different aspects of life such as education, employment and mental well-being (6) Barkley (7) argued that inhibition is the central EF impairment in ADHD, that hinders the utilisation of other functions. Moreover, a body of research reports significant deficits in the EFs of shifting and working memory for ADHD adults (8–10). Furthermore, Bailey & Jones (11) argued that the EF processes of inhibition, updating, and shifting are closely linked to emotional regulation. Henceforth, ADHD is also described as a disorder of emotional dysregulation (12). In a systematic review by Soler-Gutiérrez et al. (13), adults with ADHD demonstrated the consistent use of non-adaptive emotion regulation strategies when compared to controls. Bodalski et al. (14), also reported emotion regulation deficits in adults with ADHD including the use of avoidance strategies. Adults with ADHD demonstrate increased use of experiential and cognitive-behavioural avoidance strategies which mediates the relationship between ADHD, deficits in emotion regulation, and internalising disorder outcomes (14).

The National Institute for Health and Care Excellence (15) considers pharmacological treatment as the first-line treatment for adults with persisting ADHD symptoms. However, Ramsay (16) attests that individuals with ADHD who experience symptom improvement from medications still experience difficulties in academic and social functioning, due to ADHD’s high comorbidity with other psychological disorders, such as anxiety, depression, and substance abuse. For this reason, the NICE guideline (2018) recommends a structured psychological intervention in the form of cognitive-behavioural therapy (CBT) for individuals with ADHD as the first psychotherapeutic treatment of choice.

CBT is an umbrella term for a range of related therapies, including for instance cognitive therapy, behavioural therapy, and metacognitive therapy (17). These therapies share a strong commitment to developing clinical interventions grounded in empirical evidence, with CBT described as the most researched form of psychological therapy (18). The therapies encapsulated by the term CBT aim to reduce client’s experience of distress by helping the person to explore patterns in their behaviour, thinking processes and thought content, (19). Probably the most commonly practised form of non-adapted CBT in the UK, derives from a mixture of behavioural therapy principles and Beck’s cognitive therapy, to employ an active goal-oriented problem-solving approach (20). CBT is highly structured, present-oriented, and time-limited, usually lasting from 5–20 sessions (21). Typically, a CBT therapist may seek to address an individual’s cognitive distortions by challenging maladaptive core beliefs, dysfunctional assumptions, and negative automatic thoughts using techniques including Guided discovery, Socratic questioning, positive data logs, and thought records (21). Additionally, CBT therapists may employ behavioural techniques such as activity scheduling, where tasks are reduced to a controllable list, or behavioural experiments to try responding differently to identified situations or stimuli. The CBT therapeutic relationship is based on genuineness, rapport and empathy between the patient and the therapist (21).

In England, CBT is predominantly provided through the National Health Service (NHS) Increasing Access to Psychological Therapies Programme (IAPT), recently rebranded as NHS Talking Therapies for Anxiety and Depression (NHSTTAD). This programme was developed in 2008 in an attempt to radically increase the availability of CBT in primary care, in response to a range of NICE guidelines increasingly recommending CBT and other psychological therapies as the first-line interventions for anxiety and depression (21, 22). This programme commissions a range of primary care psychological therapies services across England, with one-to-one CBT the most frequently provided therapy (23). Therapists are trained in accordance with a competency-based curriculum (24), which does not include specific content on adapting CBT for ADHD. This potentially leads to therapists having high variability in their knowledge, skills and ability to support ADHD patients. Access to NHSTTAD services is often by self-referral, with no separate formal diagnostic assessment of presenting problems required as a precursor to treatment. While the NHSTTAD programme is mainly designed for individuals with mild to moderate depression and anxiety, therapists working in NHSTTAD services often find they are working with complex cases, for which they may have insufficient training and knowledge (22) including ADHD. According to Ramsay (4, 25), individuals with ADHD often seek treatment for comorbid depression and/or anxiety, therefore they may be highly likely to receive CBT treatment through the NHSTTAD service. Whilst statistics of the number of people accessing NHSTTAD who have an existing ADHD diagnosis, or who experience ADHD-related difficulties are not recorded, more than thirty-three thousand people seeking help from NHSTTAD services during the year 2021–22, were assessed as experiencing problems with memory, and concentration, learning and understanding (26).

Previous evidence from empirical studies reported that adults with ADHD found adapted CBT helpful for their symptoms (27, 28). Virta and colleagues (27) reported a pilot RCT of short-term outpatient adapted CBT to adults with ADHD (n=10), delivered over 10 weekly appointments. Participants in this study reported significantly reduced symptoms as a result of engaging in adapted CBT. Two patients (20%) dropped out of adapted CBT. Solanto and Scheres (28) reported a cohort study of adapted CBT for college students (n=18) delivered in a group format, over 12 weekly sessions. Clinician’s ratings and participants’ self-report data evidenced a reduction in ADHD symptoms and student’s perceived self-efficacy in managing ADHD. One participant dropped out of group adapted CBT. These studies suggest that adapted CBT is acceptable to ADHD patients.

Numerous studies have also highlighted the efficacy of adapted CBT in reducing symptoms of ADHD and EF (27–31) as well as mental well-being and general functioning (32, 33). A randomised controlled trial by Safren et al. (34) highlighted the efficacy of an ADHD-adapted CBT treatment in providing significantly better outcomes for participants over an active control treatment based on relaxation and educational support. Additionally, studies comparing CBT to treatment as usual control groups, have shown the treatment’s efficacy compared with medication-only groups (35, 36). A meta-analysis by Knouse et al. (37) reported that studies with active control groups indicated significantly smaller effect sizes for CBT treatment, than studies without active controls. The differences in these results could be due to variations in the CBT interventions applied in each study, which varied by treatment type, format, length, and the medication status of the participants, which can arguably moderate the effect of treatment (37). Finally, Solanto and Scheres reported the effectiveness of a CBT program in reducing inattention and EF in college student with ADHD.

Additionally, there are a number of studies which have shown the efficacy and acceptability of adapted Dialectical Behaviour Therapy (DBT) for ADHD patients (38–43). DBT (44) is an empirically validated approach for working with distress tolerance and coping behaviours. Early DBT papers focused on reducing self-harm and suicide attempts in individuals diagnosed with personality disorder (45), whereas more recent work has applied modified DBT to diagnostically heterogenous groups (46). DBT is often considered part of the ‘third wave’ of CBT, given its focus on emotional and behavioural regulation (47). As applied to ADHD treatment, adapted DBT includes acceptance, mindfulness, functional behavioural analysis, psychoeducation and distress tolerance techniques (42, 43). Many of the studies of adapted DBT for ADHD, have utilised group level interventions (38–43). The reliance on group interventions is at odds with the dominant model of one-to-one CBT used within NHSTTAD services. Furthermore, within the English context, DBT is a psychological therapy approach rarely delivered within primary care in England, given low numbers of DBT trained therapists and supervisors. The English NHS has plans to rapidly expand the availability of DBT by commissioning additional training (48), but there are still few DBT trained practitioners working with primary care populations.

Moreover, it is important to note that the majority of studies reporting on the efficacy and acceptability of CBT, have delivered ADHD adapted DBT or adapted CBT, rather than generic CBT, which is essential for treatment efficacy but the title and often content of these studies do not always reflect this important nuance. Ramsay (4) suggested the adaptation of CBT to accommodate for the executive and emotional dysfunctions experienced by adults with ADHD, using environmental engineering and EF training. This entails changing work, home, and personal settings by implementing systems to lessen dysfunction as well as delivering organisation and time management skills, (4). As adults with ADHD often have a history of negative experiences related to their EF deficits, which may foster negative cognitions about themselves or their abilities and maladaptive emotional strategies, these must be addressed in CBT to motivate change and encourage appropriate coping (4, 19).

Knouse & Ramsay (49) argued that non-adapted CBT could be harmful to adults with ADHD, as negative experiences of therapy can occur in relation to the experience of therapy in interaction with ADHD symptoms and individuals’ sense of self. While the benefits can outweigh the negative experiences, therapists must be aware of the possibility of certain negative experiences which might occur during all stages of a CBT treatment course, and any such experiences of therapy must be managed appropriately to reduce harm and barriers to treatment.

CBT therefore appears an efficacious treatment for people with ADHD, yet one that could cause side effects, or iatrogenic harm, if not delivered in a way that is responsive to the needs of people with ADHD. However, the existing literature provides limited indepth, qualitative insight as to how adults with ADHD experience and perceive CBT treatment. In response to this gap in the literature, the present mixed-methods study aims to record and collate the CBT experiences (adapted or non adapted) of adults with ADHD, to capture and analyse the perceived impact of this form of therapy and its value for ADHD individuals. A mixed-method approach lends itself well in capturing user experiences and understanding social phenomena better (50). This study aims to explore the following research question, ‘How do individuals with ADHD experience CBT therapy in the UK?’

## 2 Methods

### 2.1 Design

An explanatory sequential mixed methods design (51) was employed, consisting of an online survey, followed by in-depth, semi-structured interviews with a sub-sample of survey respondents. The survey data was collected over 3 months (June-August) in 2023. Interviews were conducted and recorded over one month in August 2023. The survey and interviews took place online and followed data protection procedures and best practices for record-keeping, and storage of personal data, in accordance with the BPS Code of Human Research Ethics (52). The study received ethical approval from the University of Nottingham School of Psychology (ethics reference number: FMHS 81–0922).

### 2.2 Material

The survey and interview questions were developed by the authors (who include CBT practitioners and researchers). The surveys took on average 15 minutes and included 28 questions in the form of multiple choice, 10-point Likert-scale, and free text box questions (**Supplementary Material 1**). A demographic questionnaire gathered demographic data from the samples. On average, the interviews lasted for 30 minutes and encompassed 23 questions exploring the participants’ experience of CBT and its effectiveness in addressing their ADHD difficulties (**Supplementary Material 2**).

### 2.3 Participants

Participants were recruited from across different regions of the UK, using a database of adults with a diagnosis of ADHD, collated at the University of Nottingham’s ADHD research lab. The database had been created from previous research studies with individuals who have an ADHD diagnosis who previously indicated a willingness to participate in future research studies. Additionally, participants were also recruited from, ‘The ADHD Collective’, an online community of adults with ADHD based in the UK.

Inclusion criteria were that participants were aged 18 years old or greater, had an existing diagnosis of ADHD before receiving CBT, and the course of CBT was delivered within the UK by any provider (NHS, private or others).

Participants who reported receiving CBT within a mixed, integrative or eclectic psychotherapeutic approach, such as those mixing CBT concepts with other concepts drawn from other psychotherapy approaches (e.g. psychodynamic or humanistic approaches), were excluded from the study.

### 2.4 Procedure

Details of the studies were sent to mailing lists by the research team. Participants in the survey were entered in a £10 Amazon voucher prize draw. Additionally, interview participants were provided with a £20 Amazon voucher code after the completion of the interview.

Participants in both the survey and interviews who wished to participate signed an online consent form. Participants who responded to the semi-structured interview invitation were interviewed over Microsoft Teams at a time of their convenience.

### 2.5 Analysis

The interviews were analysed using an inductive approach to thematic analysis (53), which employed an essentialist perspective in extracting codes. The thematic analysis consisted of a six-stage process (53). The analytic process began by transcribing each interview verbatim shortly after being conducted. Following this process, the lead investigator first familiarized herself with the interview data and made notes in a diary of preliminary thoughts on the content of the interviews. From this, initial codes were identified in a coding manual that was then collated and combined to be classified into broader themes using constant comparative analysis, both within and between transcripts. Finally, as the analysis evolved, these broader themes were reviewed and refined to generate the final themes proposed. An ongoing analysis allowed for a clear definition of the final themes. Semantic themes were developed using participants’ descriptions of their own experiences. Themes were then reviewed by a second researcher (BF) to ensure that they mapped to the original transcripts. Interrater reliability of themes was tested on a small proportion (2/10, 20% of interviews) of the transcripts. The results were validated collectively as a team, and any discrepancies were discussed and reconciled. The survey responses were reported descriptively and were used to triangulate the responses from the interviews.

## 3 Results

Ten participants took part in the interviews (70% female) and 46 in the surveys (71% female). **Tables 1**, **2** (Interview) and 2 (survey) describe the demographics of each group.

**Table 1:** Interview participants demographic characteristics.

	Interview participants (n=10) range (mean)
Age (years)	21–59 (43.4)
Gender (total)	
Female	7
Male	3
Number of CBT course	1–3 (1.5)
Number of CBT sessions	4–30 (13.1)
Years since CBT course	0–10 (2.8)
Years since ADHD diagnosis	01–13 (6.05)
Institution offering CBT (total)	
NHS	4
Independent provider	6

^Data is range, mean unless otherwise stated. CBT, cognitive behavioural therapy. NHS, national health service.^

**Table 2:** Survey participants demographic characteristics.

	Survey participants (n=46) range (mean)
Age (years)	20–60 (39.9)
Gender (total)	
Female	33
male	13
Number of CBT course	1–8 (1.5)
Years since ADHD diagnosis	0.5–38 (5.2)
Number of CBT sessions (total)	
Less than 6	17
6–8	11
9–12	6
More than 12	9
Unsure	3
Did you complete the CBT course (total)	
Yes	25
no	21
Institution offering CBT (total)	
NHS	23
Independent provider	18
Unsure	5

^Data is range, mean unless otherwise stated. CBT, cognitive behavioural therapy. NHS, national health service.^

### 3.1 Semi-structured interviews

The codes from the thematic analysis captured three main themes: The complex structure of the CBT framework, the intricacy of the therapist relationship, Consequences of CBT.

#### 3.1.1 The complex structure of the CBT Framework

Participants reported that the overall framework of CBT was unhelpful due to several factors. Firstly, the generic nature of CBT sessions was usually not adapted to individuals with ADHD, making therapy ineffective and experienced as highly frustrating. Secondly, the CBT sessions followed a rigid structure that was not personalised to the participants’ needs. Thirdly, the timeframe of the therapy was experienced as too short to be of benefit to the ADHD participants.

Participants reported that the CBT they received was essentially incompatible with their experience of ADHD, as it did not take into consideration the inherent EF and emotional dysregulation difficulties they experienced. Working memory deficits were not accommodated in sessions, leading to a cycle of unnecessary pressure and ineffective treatment. Moreover, participants described that the content of therapy did not account for ADHD symptoms of inconsistency, distractibility, and inattention. As a result, ADHD participants reported feeling overwhelmed and frustrated by the approach, which they found unhelpful in managing their ADHD difficulties.

*“I think there’s core things about CBT that are just seen on the face of it to me to be incompatible with ADHD. So, there is an element of having, decent working metacognition, working memory and things like that [ … ] I might discuss a technique with my therapist, but I would not remember to remember that technique. It just wasn’t going to happen.” (P5).*

Only one participant reported receiving adapted CBT, with a therapist who also had ADHD. This participant reported that their CBT sessions allowed for self-acceptance of their EF difficulties, which moderated their approach to facing ADHD-related difficulties. For instance, they were able to moderate their time and chunk activities to avoid resistance and boredom. Overall, through the adapted CBT course, they were able to adopt cognitive strategies in their daily life, easing their day-to-day activities.

In contrast, however, most participants reported that the goals set in generic CBT were unspecific and unhelpful in managing ADHD symptoms. They explained that there was often no obvious relation between the CBT process and the management of their ADHD difficulties. They reported that ADHD topics such as understanding ADHD, time management, organization, and emotion regulation were often not discussed.

“*In the sense of actually managing ADHD symptoms [ … ]like time management, procrastination, achieving goal, it wasn’t really helpful for that kind of stuff, which is initially what I was hoping for” (P3)*.

Furthermore, participants commented on the learning aids or physical resources offered in sessions. Some participants reported an absence of any learning aids or physical resources to summarize sessions, which caused an unhelpful dependence on memory, that led to forgetfulness. Conversely, other participants reported that they received an overwhelming amount of generic CBT resources which required high levels of literacy and concentration to comprehend, and which were not adequately adapted to ADHD individuals.

*“I got sent a whole load of files and stuff to read and it was just volumes and volumes and volumes and stuff [ … ] Reading stuff is something I don’t do very well, and just the thought of doing all of that just overwhelmed me. I kept losing them as well” (P6)*.

Participants reported that they needed CBT to offer an acceptance and management of their ADHD condition, rather than a fixing of their condition. Some participants reported that the sessions were too focused on symptom reduction, which did not allow for an appreciation of their strengths. This focus on just part of the person’s experience was sometimes experienced as unfair, with elements of their identity as a person with ADHD being ignored, or repressed, akin to being ‘dampened down’.

Conversely, the one participant who received adapted CBT reported that this course explained the behavioural irregularities as well as the strengths of having ADHD, fostering their acceptance of the condition.

*“What I liked about it was that I understood how my mind worked [ … ] So it was really kind of understanding what the strengths I think of ADHD were. I just felt that I’m more accepting of myself and I’m more aware of myself and I’m more aware of my kind of behaviours if that makes sense” (P4)*.

Participants also reported that the CBT objectives were not focused on the client’s needs but followed an unhelpful systematic approach. Participants who had undergone multiple courses of CBT reported that sessions felt like a pre-written script. Moreover, other participants reported that the CBT approach did not view the participant as an individual requiring personalised treatment.

*“I felt the therapist had got their own set of exercises both times that they wanted to do from their own training, and I felt that I needed a much more bespoke approach” (P9)*.

However, one participant expressed that their adapted CBT course was personalised in relation to their current situational difficulties, rather than being a generic application of CBT strategies. They reported sessions not being highly structured or systematic, but rather following an organic and client-centered approach, where the direction and flow of the therapy coincided with their feelings and needs.

Participants also reported that the generic CBT courses were too short to be helpful for their ADHD. They described that the number of offered sessions was inappropriate for individuals with ADHD who require more time to process information.

*“It’d have to be extended because not only are you meeting someone new … you still got to bring the courage to open up to that person and then the sessions end, don’t last long enough, and then the overall course doesn’t last long enough. And I feel like something that takes that much would need to have more time for it” (P7)*.

#### 3.1.2 The intricacy of the therapist relationship and its impact on therapy

Participants reported multiple difficulties with their therapists affecting the overall experience. Firstly, almost all therapists were reported to be unspecialised in working with ADHD symptoms and seemed to have little knowledge about the condition, demotivating participants. Secondly, many therapists were experienced as unempathetic, affecting the participants’ healing and learning. Thirdly, many participants described their therapists’ approach as non-accommodative and inflexible.

Therapists appeared to lack a genuine understanding of ADHD, which affected participants’ treatment and motivation to continue with therapy. Some participants commented that they believe therapists with extensive ADHD experience should be delivering the CBT to ADHD individuals, for it to be maximally effective. Several participants reported that they had to explain multiple times to their therapists that the techniques they were assigned would not work with their ADHD, creating a lack of being understood and their experiences invalidated. Additionally, participants reported that their therapists seemed to assume their mental health difficulties could be treated in the same way as neurotypicals, disregarding that the myriad difficulties participants experienced were intricately linked to ADHD.

*“I couldn’t see the link with ADHD and she didn’t see it either. [ … ] She knew nothing [about ADHD], and she told me that straight away. So, I think it impacted every single aspect of the therapy because she would just look on the surface of the problem and never be able to understand the deeper-rooted issues and difficulties” (P8)*.

In contrast, tailored CBT facilitated participants understanding of the relationship between anxiety experiences and ADHD, and this was further aided by therapist’s disclosure of personal experience and knowledge of difficulties inherent in the condition.

*“I felt very comfortable with her. I felt I could be very open and felt that she understood me, which was really important. I don’t know what it would be like to have that experience with a therapist who didn’t have ADHD … but I think unless you really know somatically how it feels that might be difficult to really know what someone else is experiencing” (P4)*.

Participants reported that their therapist was unempathetic during treatment. They often felt judged and dismissed, which worsened their emotional state and affected the healing process.

*“I always felt like quite dictated, like talking at me when I feel like, no one can be healed or learn about themselves or anything if they feel like they’re being judged or talked down to” (P7)*.

Several participants felt that their therapist was not accommodating of their difficulties, nor their explicit feedback, resulting in feeling dismissed and demotivating their activation participation in CBT.

*“I was sharing things that I thought were relevant, associated with ADHD and she didn’t really embrace it. She acknowledged it and she read it and said it was interesting, but she then didn’t necessarily adapt for it. So, I felt like it was listened to but not understood and acted upon. At the end I sort of gave up sharing my thoughts, trying to prepare for it” (P6)*.

Some participants reported situations where the therapist was extremely rigid and inflexible with the timing of sessions. For instance, one participant reported that their therapist asked them to leave the room very abruptly because their time had ended, whilst they were severely distressed from recalling a traumatic event. Another participant reported that their therapist cancelled the appointment due to a five-minute bus delay.

*“The therapist changed the time and he kept scheduling times that I couldn’t make, So, in the end, he wasn’t able to accommodate the time that I had available for the sessions, he ended up just discharging me” (P3)*.

#### 3.1.3 Consequences of unadapted CBT

The majority of participants reported little gain from or feeling worse off after the course of CBT.

Participants reported feeling worse off due to lowered self-esteem, increased sense of failure, frustration with self, increased emotional dysregulation and hopelessness with the future. One participant reported that their inability to perform the required techniques frustrated them greatly and lowered their self-esteem. Similarly, another commented that CBT made them feel responsible for their inability to benefit from the sessions, leading to a sense of failure. Other participants felt the CBT sessions left their emotional dysregulation even worse, not knowing how else they could move forward or be helped.

*“I kept forgetting to practice, so by the time I come to the next session, they would have asked me how it went with the practice and I wouldn’t have practised, I wouldn’t have had time or I would’ve forgotten. And then it felt that if I didn’t do that, we couldn’t move forward. [ … ] So it felt like I was being punished and I couldn’t do the therapy properly because I couldn’t do those exercises” (P8)*.

Some participants also felt at times that CBT sessions were a complete waste of time for them and that the lack of available alternative treatments for managing ADHD, led them feeling hopeless for the future.

*“It was just such a waste of time for everyone, and it’s a shame, [ … ] it made me feel worse going there, and that’s not what you hope when you do therapy, you expect to feel better afterwards. But I felt worse and it’s just not very nice” (P8)*.

Conversely, Participant Four described their adapted CBT experience as,

*“… very transformational … because it really helped me to understand my mind and how to kind of work, I guess with my mind more. That made me feel happier about being me rather than trying to fit into what I believe the world sort of expected of me” (P4)*.

### 3.2 Survey

All participants completed 11 Likert-scale questions on their experience of CBT from a scale of 1 to 10, where 1 indicated ‘strongly disagree’ and 10 indicated ‘strongly agree’. The results of the Likert-scale questions are presented in **Table 3**.

**Table 3:** Experience of CBT questionnaire.

Question	Mean (SD)	Mode
My CBT therapist was knowledgeable on ADHD	4.6 (3.2)	1
My difficulties were understood and treated in the context of my ADHD	3.7 (2.9)	1
CBT was adapted to accommodate my ADHD	3.6 (2.9)	1
I was made to feel that my ADHD symptoms were my fault	4.3 (2.8)	1
My therapist took the time to understand my ADHD	3.7 (2.9)	1
Overall, my experience of CBT was positive	4.2 (2.8)	3
Overall, my experience of CBT was negative	6.1 (2.4)	8
Information about CBT and my treatment was clear and easy to understand	5.5 (2.7)	5
Information about CBT and my treatment was provided in an accessible format for me	5.3 (2.8)	5
My therapist validated my difficulties because of ADHD	4.3 (2.6)	1
I found CBT really helpful	3.8 (2.8)	1

Additionally, 41 participants responded to the remaining short-answer questions. When asked, ‘What were you hoping to get out of your CBT sessions?’ participants responded that they wanted to receive help in managing their ADHD symptoms and executive functioning and to feel better about themselves. Moreover, most participants commented that they needed help understanding their thought processes and managing their emotional regulation, anxiety, self-esteem, organization, and low motivation. In addition, many participants expressed their need for actionable tools and effective coping strategies. When asked whether the CBT sessions met these expectations, participants responded that they did not. Participants commented that they felt blamed, not understood by their therapist, and constantly needed to explain themselves. For instance, one participant replied,

*“No. ADHD wasn’t understood, and I constantly felt I had to explain why some the things being asked of me were a challenge”(P124)*.

When asked about the challenges of accessing CBT, most participants argued that the sessions were too time-consuming. In addition, some participants noted that the waiting time to access CBT was too long and did not allow the patient to choose their own therapist. When asked what accommodations were made to support the participants’ access and engagement with CBT, most participants noted that no accommodations were made. Only a few participants commented that they were alerted prior to their appointments and that they were given extra time. When asked what the participants had liked or disliked, found helpful or unhelpful about CBT, many participants responded that it was unhelpful because it was manualised, repetitive, and did not address the underlying causes of symptoms. Moreover, some participants commented that they found the homework, tools, and therapists unhelpful, increasing their frustration. For example, one participant wrote,

*“I struggled with speaking to someone who didn’t understand ADHD and didn’t seem to want to make any effort to. Some of the tasks required more forward planning or future thinking than I’m able to engage with. I came away feeling I’d need a much more intense level of interaction and support than I could afford or was on offer”(P106)*.

When asked what the CBT course included, most participants responded that the course included working on unhelpful thinking styles, managing multiple tasks, organisation and planning, and managing distractibility. Moreover, when asked whether they had anything else to add about their experiences with CBT, some participants responded that they did not find it suitable and would not recommend this form of therapy to individuals with ADHD. For instance, one participant said,

*“Overall, it made me feel more inadequate as I felt I couldn’t do the stuff I was supposed to. You can’t change how you think when your brain is wired differently. ADHD isn’t a thinking or positivity problem, and CBT seemed to assume it was”(P121)*.

## 4 Discussion

The present study aimed to explore how individuals with ADHD experienced CBT in the UK. In this study, individuals with ADHD experienced several difficulties with CBT, that was not adapted to ADHD, which could have a negative impact on their overall wellbeing. These difficulties encompassed nonalignment of an unadapted CBT framework with specific aspects of ADHD, alongside a perceived unspecialised, unempathetic and non-accommodative CBT therapist, collectively resulting in suboptimal therapeutic experiences.

Participants expressed frustrations with the generic CBT framework due to its inconsideration of the EF and emotional dysregulation impairments experienced by individuals with ADHD. Participants described being forgetful, distracted, inconsistent, and inattentive, which pertained to impairments in their EF processes of updating, shifting, and inhibition, supporting previous research highlighting these difficulties in ADHD adults (8–10). Moreover, the participants’ emphasis on emotional regulation difficulties further supports previous research describing ADHD as a disorder of emotional dysregulation (14, 54). Sadly, the generic, non-adapted CBT framework was not experienced as helpful, causing a counterproductive effect where participants felt overwhelmed, frustrated, and hopeless.

Research shows that when CBT is adapted specifically for ADHD symptoms, it can provide concrete strategies for managing the core symptoms of inattention, hyperactivity and impulsivity, and the associated personal interpersonal, social and occupational concomitants of the condition (55). Additionally, adapted DBT group interventions have demonstrated high effectiveness and acceptability, in helping people manage ADHD related symptoms (38–43). Group delivery of therapy is not commonplace within NHSTTAD services for patients with higher levels of distress or complexity, with one to one CBT being the primary treatment option. Moreover, as previously highlighted, there are few DBT trained therapists and supervisors currently working in primary care within England, giving rise to current plans to increase numbers of DBT trained therapists (48). The implication is that at this present time, adapted DBT maybe unlikely to be delivered in primary care with fidelity to the empirical studies.

Hayes and Hoffman (47), make the point that ‘third wave’ and traditional CBT approaches are often blended in reality, and this may be reflected in the range of empirically validated key adaptations to CBT for ADHD, which include helping the person to develop and review strategies to improve attentional focus, impulse control, planning and problem-solving, cognitive restructuring in the context of ADHD, managing emotional arousal in conflict and ensuing emotional or behavioural responses (e.g. managing anger and anxiety) and pro-social skills, e.g. empathy skills including perspective taking, recognition of the thoughts and feeling of others, critical reasoning, evaluating options and negotiation skills (28, 34, 56).

This is consistent with a body of research showing the efficacy of CBT in reducing ADHD symptoms and improving EF (29, 31, 34, 56). Moreover, in a recent meta-analysis by Young et al. (19), CBT was shown to be an effective psychotherapeutic treatment for reducing ADHD symptoms.

Potential inconsistency in results across included studies is affected by stark differences in the implementation and delivery of CBT. Ramsay (4) described the impeding effect of ADHD symptoms on standard CBT and the need for an adapted approach to CBT to accommodate the EF and emotional dysregulation difficulties in participants with ADHD. Additionally, previous studies reported CBT content targeted to address ADHD symptoms, in countries outside the UK (19, 31, 34, 56, 57). The English NHSTTAD system is unique as it is a single point of access for CBT for all resident adults seeking support with mental health, following a prescribed competency-based approach to CBT for a limited range of presenting problems (58). Therefore, CBT in NHSTTAD is not necessarily easily tailored to or adapted for specific conditions outside of its core focus on anxiety and depression. CBT programs in other countries and published studies have often been adapted for ADHD and therefore do not represent the same form of care.

The difference in outcome between adapted and generic CBT is demonstrated in the striking disparity between Participant Four’s account and those of the other participants. They received a form of CBT specifically adapted for individuals with ADHD, by a therapist who was reported as having specialist expertise in working with clients with ADHD and who also had lived experience of ADHD. This experience of CBT was found extremely helpful and meaningfully tailored to their experiences by explaining their cognitive processes and behavioural responses in the context of their ADHD diagnosis. Psychoeducation of ADHD and an adapted approach allowed for an understanding of the client’s strengths and promoted self-acceptance and moderation of their ADHD-related difficulties. This mirrors previous studies which have highlighted the benefits of psychoeducation in cognitive interventions (43). Conversely, most participants, reported that there was no obvious accounting for ADHD symptoms within their CBT sessions. Therapists appeared to lack cursory knowledge of ADHD and did not seem to understand ADHD as a root cause behind symptoms experienced, and therefore could not appropriately adapt CBT or provide relevant techniques to help clients accept and moderate ADHD-related difficulties. Similar experiences of CBT delivered in routine practice in NHSTTAD services, as not being adequately tailored to the needs of clients are reported in the literature. Omylinska-Thurston et al. (59) reported similar findings in a group of participants with severe mental health disorders, where generic CBT was not experienced as adequately addressing underlying core issues, and was delivered inflexibly, leading to CBT being perceived as a waste of time and financial resources. The pressure on NHSTTAD therapists is significant, including considerations such as measurement against key performance indicators relating to client and service recovery rates, ‘throughput’ of clients, limited session numbers, high caseloads, and a range of client problems that are less likely to respond to time-limited CBT, such as experiences of poverty, social exclusion, or systematic oppression and social injustice (22). Against such a demanding context, several studies report significant levels of stress and psychological disturbance among the NHSTTAD workforce (60–62). It is possible, that against this context of background stress, therapists may be struggling to provide personalised formulation and therapy adapted to the presenting needs of their clients.

Indeed, in this study, most participants reported not receiving behavioural components of CBT for ADHD, meaning that they were not given graded task assignments, activity scheduling, or other behavioural tools to help manage procrastination and anxiety. The exclusion of valid behavioural elements of CBT has been previously noted by Binnie (22), who argued that CBT delivered in NHSTTAD often tended to focus on cognitive interventions, neglecting valid behavioural components.

Participants argued that the structure of therapy was not client-centred but followed a rigid and systematic approach which neglected their feelings, needs, and self-expression. Decades of research highlight the importance of a therapeutic relationship in which the therapist is experienced as empathic and attuned to the needs of the client, (e.g. 63), however, this crucial element of therapy was not experienced by several participants in the present study. Omylinska-Thurston et al. (59) reported that when participants felt their therapists were unempathetic and adhered to a rigid CBT protocol, instead of attending to the participant’s individual needs, therapy was unhelpful. Binnie (22) supported this by arguing that the delivery of CBT in NHSTTAD services may omit collaborative empiricism and guided discovery where the therapist works compassionately with the client, and instead overly focuses on manualised treatment for a restrictive range of presenting problems.

In contrast, Participant Four’s, specialised therapist idiosyncratically formulated the participant’s current situational difficulties and meaningfully personalised the treatment plan to the participant’s feelings and needs. This was experienced as crucial and helpful by the participant, who was able to learn from and manage undesirable situations, supporting Omylinska-Thurston et al. (59) who argued that an adjusted client-centred (i.e. idiosyncratically formulated) CBT process can improve the therapeutic relationship and outcome of therapy.

Overall, most participants reported feeling discontent or disappointed with therapy, which led to an increased sense of failure, increased emotional dysregulation, low self-esteem and a sense of self-blame. The ineffectiveness of therapy increased their feelings of hopelessness and disappointment in themselves. According to Ramsay (4), individuals with ADHD are more inclined to have pessimistic thoughts and expectations of failure due to their past unsuccessful experiences, which runs the risk of being amplified by therapy not adjusted to consider the person’s experiences of ADHD.

The survey results further supported the insights gleaned from the conducted interviews. Similar to the interviews, participants responded that they found the non-adapted form of CBT unhelpful and challenging, further deploring their self-esteem and increasing their frustration. Moreover, the therapists’ lack of knowledge of ADHD was apparent from most survey responses, demonstrating a need for additional training for therapists, on working with people who have ADHD.

### 4.1 Limitations

While the present study addresses an important research gap on the experience of generic, non-adapted CBT in adults with ADHD, there are limitations to the study. A convenience sample was used to recruit participants. The sample was predominantly female, which may not be an adequate representation of the predominantly male ADHD population, limiting the generalisability of the results. Moreover, convenience sampling may attract participants with charged emotional experiences, who may deliver a more negatively, or positively exaggerated account than that of the rest of the ADHD population. Additionally, the impact of the different ADHD presentations (inattentive, hyperactive-impulsive, and combined) on participants’ experiences of CBT was not analysed, which may have left an interesting variable unexplored. Finally, it is important to acknowledge that the findings refer to a vast range of non-adapted CBT treatment episodes experienced across the UK and therefore refers to a heterogeneous form of therapy. While we could discern between private, adapted CBT programs and NHS delivered generic programs, we cannot generalise the findings broadly as we lack details on these specific programs. Finally, we did not explore the different types of CBT that might have been received. The study aimed to look into how adults with ADHD experienced CBT, adopting a broad definition of what CBT is, as we did not want to be too prescriptive, believing that individuals might not always know the exact type of CBT they have received. This variance in the nature of CBT delivered, and understanding of what type of CBT is received may reflect naturalistic practice in the NHS, however through this omission, we might have missed important information about different nuances.

### 4.2 Future considerations

#### 4.2.1 Implications for practice

This study highlights that routine delivery of CBT in the UK, may not be adapted appropriately for many adults with ADHD, negatively impacting their experiences. To combat this counterproductive effect of therapy, CBT therapists treating ADHD adults must receive additional training on adapting CBT to work with the array of symptoms and common experiences of people with ADHD, to more appropriately adapt CBT techniques and resources (4). Through this adaptive framework, necessary considerations regarding the EF and emotional dysregulation difficulties of ADHD individuals should be considered, transforming the nature of standard CBT to being more explicitly aligned with the experiences of people with ADHD.

#### 4.2.2 Implications for research

The present study illustrates the potential negative impact of CBT on adults with ADHD revealing the need for more research in this topic area. Further investigation on the difference between adapted versus non-adapted CBT would further the important nuance in how beneficial CBT may be as a first line of psychotherapy treatment. Additionally, future research should consider the effect of different ADHD presentations on the effectiveness of CBT treatments, since research suggests improvement for clients with the predominantly inattentive ADHD sub-type (64). Moreover, specific post-qualification training on adapting CBT to work with ADHD symptoms appears indicated, and the authors are developing such training packages in association with people with lived experience of ADHD.

## 5 Conclusion

In conclusion, the present study portrays how adults with ADHD experienced CBT in the UK, with most ADHD participants reporting negative experiences when CBT programs were not adapted. This evidence prompts future research and clinical practice to address the issues highlighted in this study for a deeper understanding of how best to accommodate adults with ADHD in therapy. Moreover, this prompts therapists and service providers in the UK to consider the current implementation of CBT to ensure CBT can be appropriately adapted and delivered by therapists with relevant training, who understand the difficulties of ADHD, to ensure that treatment is helpful, efficient and meaningful to adults with ADHD, and to mitigate against the possibility of iatrogenic harm.

## Data availability statement

The raw data supporting the conclusions of this article will be made available by the authors, without undue reservation.

## Ethics statement

The studies involving humans were approved by University of Nottingham School of Psychology ethics committee (ethics reference number: FMHS 81-0922. The studies were conducted in accordance with the local legislation and institutional requirements. The participants provided their written informed consent to participate in this study.

## Author contributions

SW: Formal analysis, Investigation, Writing – original draft, Writing – review & editing. MH: Conceptualization, Methodology, Supervision, Writing – review & editing. JR: Conceptualization, Methodology, Supervision, Writing – review & editing. CH: Conceptualization, Investigation, Supervision, Writing – review & editing. BF: Conceptualization, Investigation, Methodology, Project administration, Supervision, Writing – review & editing.
