# Supplementary material for: Experience of CBT in adults with ADHD: a mixed methods study
Source: Front Psychiatry. 2024 Jun 19;15:1341624. doi: 10.3389/fpsyt.2024.1341624 (PMC11221408; doi:10.3389/fpsyt.2024.1341624)
Supplement: Supplementary file 1 [file DataSheet_1.zip › Supplementary Material 1.DOCX]

# Survey

## Demographic

Age:

Gender:

Ethnic background:

Years since ADHD diagnosis:

Did you receive CBT to help with ADHD difficulties?

If you received CBT to help with ADHD difficulties (if you answered 'yes' to the question above), when was CBT suggested/prescribed to you? (For example: CBT was suggested after I received my ADHD...)

If you have received a CBT course more than once, please indicate how many times.

Did you complete the course of CBT you started?

How long did it last?

Was this offered through the NHS or an independent provider?

## Experience of CBT

**Instructions: On a scale of one to 10 ( 1 being strongly disagree and 10 being strongly agree), how would you rate the statements below**

My CBT therapist was knowledgeable on ADHD

My difficulties were understood and treated in the context of my ADHD

CBT was adapted to accommodate my ADHD

I was made to feel that my ADHD symptoms were my fault

My therapist took the time to understand my ADHD

Overall, my experience of CBT was positive

Overall, my experience of CBT was negative

Information about CBT and my treatment was clear and easy to understand

Information about CBT and my treatment was provided in an accessible format for me

My therapist validated my difficulties because of ADHD

I found CBT really helpful

**instructions: We would like to learn more about your experience of CBT. Please answer the questions below with as much details as possible.**

What were you hoping to get out of your CBT sessions?

Did the CBT sessions meet your expectations?

What challenges did you experience accessing CBT?

What did you like or dislike, find helpful or unhelpful, about CBT?

What accommodations, if any, were made to support your access & engagement with CBT?

Do you have anything to add about your experience of CBT?

Did your CBT course include any of the following? (Select all that apply)

- Education about ADHD
- A formulation (a shared understanding with your therapist about the origin, current status, and maintenance of the difficulties you were seeking help for)
- Working on managing multiple tasks, organising and planning
- Managing distractibility or procrastination
- Working on unhelpful thinking styles
