# Supplementary material for: Experience of CBT in adults with ADHD: a mixed methods study
Source: Front Psychiatry. 2024 Jun 19;15:1341624. doi: 10.3389/fpsyt.2024.1341624 (PMC11221408; doi:10.3389/fpsyt.2024.1341624)
Supplement: Supplementary file 1 [file DataSheet_1.zip › Supplementary Material 2.DOCX]

**Interview Questions**

General Info

1. What is your experience of CBT?
2. What did you access CBT for? Private or NHS?
3. How long after your diagnosis of ADHD did you access CBT?
4. If you did not access CBT directly for your ADHD, did you inform your therapist about your ADHD diagnosis before starting CBT for the other mental health condition?
5. Were any accommodations made available to support your access and engagement with CBT? Are there any accommodations that you think would have better enabled your access and engagement with CBT? (Ex: allowed extra time/ allowed to be a little late

CBT-related Questions

1. What did you like or find helpful about CBT?
2. Anything specific for your ADHD?
3. What did you dislike or find unhelpful about CBT?
4. Anything specific for your ADHD?
5. What were you hoping to get out of your CBT sessions?
6. How well did your therapist or mental health professional explain the CBT process and its potential benefits for managing your ADHD?
7. Did you feel actively involved in setting goals and designing the treatment plan during your CBT sessions for ADHD?
8. Did the CBT sessions meet your expectations?
9. Do you have anything to add about your experience of CBT?
10. Did you find the techniques and strategies learned in CBT helpful in your daily life?

Please describe.

ADHD-related Questions

1. Did your therapist explain what ADHD is?
2. Did your therapy include specific ADHD topics such as understanding ADHD, time management, organisation, emotion regulation etc.?
3. Did you think your therapist was knowledgeable about ADHD?

If not, could you give examples on how this was experienced?

1. Did you feel that your difficulties related to ADHD were understood by your therapist?

If not, could you give examples on how this was experienced?

1. If your therapist was not familiar with ADHD, what did they do ' e.g., asked me for further information on ADHD or how it affects me, researched ADHD further themselves, sought ADHD-specific consultation, referred me elsewhere?’
2. What specific ADHD symptoms or challenges do you believe CBT has helped you address?
3. Did you feel you had a bond with your therapist/or that it was a 2-way interaction?
4. How did you feel after the CBT? Neutral? Better-off? Worse-off?
